# Clinical outcomes of an intraoperative surgical margin assessment using the fresh frozen section method in patients with invasive breast cancer undergoing breast-conserving surgery – a single center analysis

**DOI:** 10.1038/s41598-019-49951-y

**Published:** 2019-09-17

**Authors:** Tomasz Nowikiewicz, Ewa Śrutek, Iwona Głowacka-Mrotek, Magdalena Tarkowska, Agnieszka Żyromska, Wojciech Zegarski

**Affiliations:** 1 Chair and Department of Surgical Oncology, Ludwik Rydygier’s Collegium Medicum UMK in Bydgoszcz, Prof I. Romanowskiej 2, 85-796 Bydgoszcz, Poland; 2 Department of Clinical Breast Cancer and Reconstructive Surgery, Oncology Centre-Prof. Franciszek Łukaszczyk Memorial Hospital, Prof I. Romanowskiej 2, 85-796 Bydgoszcz, Poland; 3 Department of Rehabilitation, Ludwik Rydygier’s Collegium Medicum UMK in Bydgoszcz, M. Sklodowskiej-Curie 9, 85-001 Bydgoszcz, Poland; 4 Department of Laser Therapy and Physiotherapy, Ludwik Rydygier’s Collegium Medicum UMK in Bydgoszcz, M. Sklodowskiej-Curie 9, 85-001 Bydgoszcz, Poland; 5 Chair and Clinic of Oncology and Brachytherapy, Nicolaus Copernicus University Ludwik Rydygier Collegium Medicum in Bydgoszcz, Prof I. Romanowskiej 2, 85-796 Bydgoszcz, Poland; 6 Amethyst Radiotherapy Centre, Zgorzelec, Poland

**Keywords:** Breast cancer, Cancer imaging, Surgical oncology

## Abstract

Breast conserving treatment (BCT) is a safe standard therapeutic method in patients with early invasive breast cancer. However, it is associated with an increased risk of residual neoplastic tissues in surgical margins. The aim of this study was to assess the outcome of the use of the intraoperative pathologic analysis by the frozen section (FS) method for evaluation of the extent of the primary lumpectomy. The study concerns a retrospective analysis of a group of 1102 patients who underwent BCT between Jan 2015 and Dec 2016. The assessment focused on the frequency of the intraoperative pathologic analysis of the primary lumpectomy extent (fresh frozen section method). The influence of the BCT specimen analysis method on the free margins width, as well as the rate and the cause of reoperation were evaluated. The intraoperative lumpectomy evaluation was performed in 45.8% (505/1102) of patients (Group I), while in the remaining 54.2% of the cases it was decided to abandon this procedure (Group II). Although in 72 (14.3%) patients the intraoperative analysis gave negative results, the margins contained residual tumor tissue (vs. 16.9% in Group II). In Group I, conversion from the previously planned BCT to mastectomy was necessary in 5.9% (30/505) patients (vs. 9.7% in Group II). The duration of surgery was 48.9 ± 17.3 minutes (Group I) and 42.9 ± 13.6 minutes (Group II). In patients undergoing BCT, the use of the intraoperative pathologic analysis by the FS method resulted in a reduction of the total number of reoperations performed due to residual tumor found in the margins following the primary lumpectomy. However, it statistically significantly extended the duration of the surgery.

## Introduction

As shown by Veronesi *et al*. and by Fischer *et al*., breast-conserving treatment (BCT) is a safe and effective therapeutic method in patients with early invasive breast cancer^1,2^. This was confirmed by results of subsequent randomized clinical trials^3,4^, which also established that, contrary to mastectomy, in patients treated with BCT the risk of residual tumor presence within surgical margins after primary tumor resection is higher^3^.

Non-radical primary tumor resection is the main risk factor for local recurrence^5^. Neoplastic cells within surgical margins are found in 20–30% of patients after local breast resection cancer^5,6^, with majority of them being subjected to reoperation^7^. It raises overall costs of treatment, as well as the rate of postoperative complications^5^.

One of the tools that can be used to evaluate the extent of the BCT procedure is the intraoperative pathologic analysis of a tissue sample, to evaluate surgical margins. The most common technique used for this purpose is a microscopic frozen-section analysis (FS). However, when compared to the final pathologic analysis of paraffin blocks, this method shows some limitations. First of all, it is characterized by considerably lower sensitivity of 65–78%, and its use extends the surgical procedure duration^8–10^.

Other diagnostic methods can also be used for the intraoperative assessment of surgical margins, including: radiology studies (mammography – MMG, ultrasound scan – USS, magnetic resonance – MRI, or *optical coherence elastography* – OCE), fluorescent techniques (using indocyanine green and IRDye800CW), optical techniques (based on the light spectrum analysis), isotopic methods (using ^111^In or ^89^Zr) and other pathologic techniques (*touch imprint cytology*, *macroscopic margin assessment*)^7,11,12^. The extent of their use depends on preferences of a given breast cancer treatment center.

The intraoperative pathologic examination of surgical margins by the FS method is not a standard procedure in patients treated by BCT. This concerns, in particular, treatment of women suffering from carcinoma *in situ*, where in those cases such pathologic evaluation of the specimen should be avoided^13–15^. However, at some oncologic centers, the intraoperative pathologic analysis of the tumor is used in the daily clinical practice. In our clinic, this examination was performed on a routine basis in the past, but currently its frequency is reduced, depending on an individual decision of a surgeon.

The aim of this study was to evaluate clinical consequences of the intraoperative pathologic analysis performed to verify the extent of the primary tumor excision in a group of patients suffering from early breast cancer, who underwent BCT. The obtained results were compared to results obtained in a group of patients whose pathologic status was established by a final pathologic analysis.

## Materials and Methods

### Studied group of patients

A retrospective analysis was performed at a reference center for specialized surgical treatment of invasive breast cancer. It used clinical data of 1102 patients with invasive breast cancer (diagnosed on a basis of core-needle biopsy of the lesion), who underwent the breast-conserving treatment (inclusion criteria). The study covered patients hospitalized at our center between Jan 2015 and Dec 2016.

Patients qualified for BCT for non-invasive breast cancers and non-epithelial malignancies, or for invasive breast cancer diagnosed by surgical biopsy of suspected lesion (tumorectomy), or requiring preoperative systemic treatment were excluded from the analysis (exclusion criteria). This study was approved by the Bioethics Committee of Collegium Medicum Nicolaus Copernicus University in Bydgoszcz (No. 235/2016).

### Analyzed clinical data

The following clinical parameters were determined in the study subjects: patient’s age, primary tumor size (cT), histological type and grade, presence of *comedonecrosis* and multifocality, additional presence of ductal carcinoma *in situ* (DCIS), estrogen (ER), progesterone (PR)^14^ and HER2 receptors status^16^, biological type of breast cancer^17,18^, and a need for simultaneous axillary lymph node dissection.

The study used clinical data from a prospectively maintained database that ensured the complete anonymity of the subjects. Hospital medical documentation of the patients was used as an additional source of information.

### Surgical techniques

All patients enrolled in this study underwent complete surgical treatment at our center. Patients were qualified for BCT according to the procedure based on generally accepted international standards of breast cancer treatment^14,15^. In accordance with the current guidelines, it required tumor resection within healthy tissues (no ink on the tumor).

**Figure 1:**
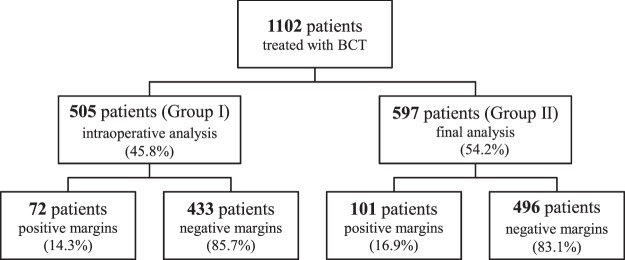
Studied group of patients.

Tissue samples obtained during BCT underwent the pathologic analysis of surgical margins. Depending on an individual decision made by a surgeon, a pathologic verification of tumor resection extent in some patients was performed intraoperatively (using the frozen section, FS, method), followed by a final assessment – Group I, or only as the final assessment – Group II (Fig. 1).

In each case, the method for examination of the excised tumor was selected according to a fully autonomic surgeon’s decision. In consequence, two groups of patients were distinguished, as mentioned above.

This study was a retrospective analysis. It was conducted at the time when our facility gradually moved away from the routine intraoperative pathologic analysis of samples obtained during BCT (initiated to shorten the duration of surgery and reduce costs of diagnostic and therapeutic procedures). This prompted us to evaluate clinical consequences of the change in the method used to evaluate tissue specimens post BCT.

Regardless of the method selected for the pathologic analysis, when nonpalpable lesions were treated, the surgical intervention was preceded by tumor labelling with a metal marker (introduced percutaneously under USS or MMG guidance). Each resection of the mass with such marker was followed by an intraoperative mammographic assessment of the specimen. USS was not used for this purpose. When there were any doubts concerning correct resection of the mass, as indicated by the radiology results, resection margins were extended during the same surgery.

Patients with palpable masses did not undergo the radiologic evaluation of the sample, according to the diagnostic and therapeutic algorithm applied at our hospital. It was replaced by the intraoperative pathologic analysis (FS – Group I), or by the gross clinical evaluation of a lumpectomy specimen performed by a surgeon (Group II). For this reason, an additional aim of the initiated study was to answer a question whether the FS method for surgical margin assessment should be replaced by a different intraoperative evaluation of the specimen (Group II).

In both distinguished groups of patients, the width of surgical margins was assessed (a value exceeding 10 mm is described as ‘greater than 10 mm’). Regardless of a type of the pathologic analysis used (the intraoperative or the final specimen analysis), the cancer lesions were resected with a margin of normal tissues that was at least 1 mm wide. The results from the postoperative pathology reports were statistically analyzed. Furthermore, the rate of non-radical resections, a frequency and type of reoperations post BCT, as well as the duration of primary intervention were compared.

Presence of cancer tissue in surgical margins after BCT resulted in extended tumor resection.

When FS examination (Group I) proved that the surgery was insufficiently radical, the additional margin (or margins) were removed during the same procedure (and they underwent only final pathologic analysis). Whereas, when tumor cells were found in surgical margins during the final pathologic analysis (Group I and II), the patients required reoperation. The type of the repeated procedure (requadrantectomy or mastectomy) depended on a result in the pathologic report and on an individual decision of a patient.

Neither the weight nor the volume of removed tissue specimens were analyzed in the studied group of patients (this information was not determined at our center on a routine basis during this study). The evaluation also did not include aesthetic outcomes of surgical intervention used in the patients.

### Pathologic analysis of the margins of the primary lumpectomy

During the operation, the breast tumor was widely excised with 1.0–3.0 cm macroscopic margin. After the resection, tumor specimens were oriented using sutures and transported as frozen sections (FS) to the pathology department for the intraoperative pathologic analysis. The outer lumpectomy specimen margins were marked with ink and the specimen was sectioned into 5-mm slices along their long axis. The tumor size and the margin width were determined. FS was performed for four margins of the tumor (superior, inferior, medial and lateral). The standard approach involved four specimens, sometimes FS was performed for lesions of unknown diagnosis (no preoperative core needle biopsy). Frozen sections biopsies measuring approximately 1.5 × 1.5 cm in size were taken. The tissue to be frozen was placed on a cryostat chuck with a tissue freezing medium (Shandon Cryomatrix) and mounted in the cryostat (Shandon Cryotome FSE). Approximately 10-μm thick sections were collected, placed on slides and stained with haematoxylin and eosin (H&E) and microscopically examined by one or two experienced pathologist. The median duration of the frozen section procedure was 15 min.

Intraoperative FS results for each biopsy were recorded as positive: invasive carcinoma, DCIS, LCIS and atypical cells. Patients with positive or suspicious margin underwent re-excision or mastectomy. Frozen sections were not done for those specimens. The breast tissue corresponded to the positive margin was marked by sutures.

After FS, the specimen underwent routine processing and was fixed in phosphate-buffered 10% formalin for 24 hours. The tissue from all margins and from a tumor was embedded in paraffin blocks and sections 4–5 mm were stained with H&E for the definitive histological analysis. A final report of the margins status was compared with the result of the frozen section analysis.

### Statistical analysis

All statistical analyses were performed with the Dell Statistica data analysis software system, version 13. Data was summarized as a mean ± standard deviation for continuous variables, or a number of patients and a percentage for categorical variables. Comparisons were made by two-sample t tests for continuous variables and by the chi-square analysis for categorical variables.

Sensitivity was defined as true-positive results/(true-positive results + false-negative results), whereas specificity was defined as true-negative results/(false-positive results + true-negative results).

A p value of <0.05 was considered to be statistically significant.

## Results

The intraoperative pathologic analysis of the specimen by FS was used in 45.8% of patients (505/1102) who underwent BCT (Group I). In remaining 54.2% of the patients, only the final pathologic tumor analysis was performed (Group II).

For the majority of the analyzed clinical features, no statistically significant differences were noted between the two groups. Only the rate of palpable masses (p = 0.0024), the number of cT2 patients (p = 0.0369), and the number of patients requiring simultaneous axillary lymph node dissection (p = 0.0124) were significantly higher in Group I (Table 1).

**Table 1:** Patients undergoing the procedure – clinical characteristics.

Clinical data	Group I	Group II	P-value
	(n = 505)	(n = 597)	
	# (%)	# (%)	
Mean age (years) and range	58.7 (25–85)	59.4 (27–87)	0.2421
BMI (mean)	27.08 ± 4.98	27.43 ± 5.08	0.2484
**Histological type**			
invasive ductal	446 (88.3%)	513 (85.9%)	0.2379
invasive lobular	42 (8.3%)	64 (10.7%)	0.1778
invasive – other types	17 (3.4%)	20 (3.4%)	0.9883
Presence of DCIS	164 (32.5%)	221 (37.0%)	0.1185
Presence of *comedo* necrosis	11 (2.2%)	13 (2.2%)	0.9994
Tumor multifocality	81 (16.0%)	113 (18.9%)	0.2075
**Tumor stage**			
T1	310 (61.4%)	395 (66.2%)	0.0981
T2	191 (37.8%)	190 (31.8%)	0.0369
T3	4 (0.8%)	12 (2.0%)	0.0968
Palpable tumor mass	357 (70.7%)	370 (62.0%)	0.0024
**Histological grade**			
G1	25 (5.0%)	27 (4.5%)	0.6968
G2	352 (69.7%)	445 (74.5%)	0.076
G3	107 (21.2%)	100 (16.8%)	0.0626
nd	21 (4.2%)	25 (4.2%)	0.9809
ER positive	428 (84.8%)	498 (83.4%)	0.5272
PR positive	385 (76.2%)	433 (72.5%)	0.1619
HER2 positive	62 (12.3%)	73 (12.2%)	0.9598
**Molecular subtype**			
luminal A	316 (62.6%)	377 (63.1%)	0.7843
luminal B HER2 negative	67 (13.3%)	74 (12.4%)	0.656
luminal B HER2 positive	46 (9.1%)	47 (7.9%)	0.4755
non-luminal HER2 positive	16 (3.2%)	26 (4.4%)	0.3023
triple negative	60 (11.9%)	73 (12.2%)	0.8789
Simultaneous axillary lymph node dissection	54 (10.7%)	39 (6.5%)	0.0124

In 501 (99.2%) patients from Group I no cancer tissue was found in surgical margins in the intraoperative pathologic analysis. In the remaining four cases, invasive cancer was found within them, and for this reason the extent of the primary tumor resection was simultaneously extended in each of these patients.

Despite a negative result of the intraoperative FS tumor analysis, presence of cancer tissue was found in surgical margins in 72 (14.3%) patients in Group I after the final pathologic analysis. 41 (56.9%) patients were diagnosed with invasive cancer, while in the remaining cases the lesions were preinvasive. In Group II, where surgical margins were evaluated only by the final analysis, this situation concerned 101 (16.9% p = 0.2373) patients (Table 2). In all these patients, the surgery was radicalized.

**Table 2:** Results of a surgical margin analysis post initial breast sparing treatment.

Clinical data	Group I	Group II	p
	n = 505 (%)	n = 597 (%)	
Negative margins (benign lesions)	433 (85.7%)	496 (83.1%)	0.1189
Positive margins	72 (14.3%)	101 (16.9%)	0.2373
- invasive cancer	41/72 [56.9%]	57/101 [56.4%]	
- DCIS	28/72 [38.9%]	40/101 [39.6%]	
- LCIS	3/72 [4.2%]	4/101 [4.0%]	
Cancer cells present in a specimen after reoperation	35 (48.6%)	58 (57.4%)	0.0959

The specimen FS pathologic analysis after BCT was characterized by sensitivity of 5.3%, specificity of 100%, a positive predictive value (PPV) of 100%, and negative predictive value (NPV) of 85.6% (the number of true-positive results, true-negative results, false-positive and false-negative results was 4, 429, 0, and 72 patients, respectively).

Requadrantectomy was performed in 58.3% (42/72) of reoperated patients from Group I (vs 42.6%–43/101 – in Group II). In the remaining patients, it was necessary to abandon the initially planned breast-conserving treatment and perform mastectomy (Table 3).

**Table 3:** Patients participating in the trial – treatment results.

Clinical data	Group I	Group II	P-value
	(n=505)	(n=597)	
	# (%)	# (%)	
Need for reoperation	72 (14.3%)	101 (16.9%)	0.2373
Need for mastectomy	30 (5.9%)	58 (9.7%)	0.0202
**Initial surgical margins width after BCT:**			
- within the range of 0–10 mm	225 (44.6%)	332 (55.6%)	0.0028
[mean]	[6.6 ± 4.7 mm]	[6.5 ± 4.6 mm]	0.7219
- 0 mm	72 (14.3%)	101 (16.9%)	
- 1 mm	1 (0.2%)	3 (0.5%)	
- 2–3 mm	1 (0.2%)	2 (0.3%)	
-4–5 mm	5 (1.0%)	19 (3.2%)	
- 6–10 mm	146 (28.9%)	207 (34.7%)	
over 10 mm	280 (55.4%)	265 (44.4%)	0.0003
Surgery duration [min]	48.9 ± 17.3	42.9 ± 13.6	<0.0001

In 44.4% of the cases from Group II the width of surgical margins after initial BCT exceeded 10 mm (vs 55.4% in group I; p = 0.0003). The results ranged from 0 to 10 mm in the remaining patients, with the mean width of 6.6 ± 4.7 mm in Group I and 6.5 ± 4.6 mm in Group II. The average duration of surgery was 48.9 ± 17.3 minutes in Group I and 42.9 ± 13.6 minutes in Group II (p < 0.0001) (Table 3).

**Figure 2:**
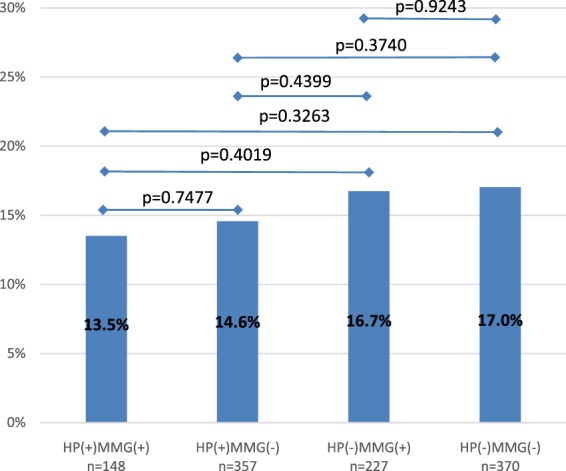
Patients qualified for the procedure – influence of an intraoperative analysis type on the positive margin rate.

Furthermore, in the studied group of patients the effectiveness of the intraoperative analyses of tumor resection margins (the mammographic specimen assessment after BCT, FS pathologic analysis) was also evaluated. Non-radical tumor resection during the primary surgery was usually performed in those cases, where none of the listed analyses was done: HP(−)MMG(−) group - 17.0% of cases. On the other hand, tumor cells in the surgical margins were most rarely found in patients in whom both discussed methods for the BCT extent analysis were used: HP(+)MMG(+) group – 13.5% of women. However, no statistically significant differences were observed between the groups analyzed in that respect (Fig. 2).

## Discussion

This study was conducted to evaluate clinical outcomes resulting from the changed approach to the analysis of BCT extent in patients diagnosed with early breast cancer, used at our center. The routinely performed intraoperative pathologic analysis of a specimen using the frozen section procedure (confirmed by microscopic evaluation of paraffin-embedded blocks) was replaced by the final analysis alone. This change led to several clinical consequences, already described in section “Results” of this paper. One of major differences between the evaluated groups of patients was the rate of positive resection margins (14.3% vs 16.9%). However, the observed differences proved not to be statistically significant (p = 0.2373).

According to the presented clinical characteristics, the study compared groups of patients with similar values for analyzed variables (excluding the rate of palpable lesions: 70.7% in Group I vs 62.0% in Group II, and the percentage of cT2 masses: 37.8% vs 31.8%, respectively). Thus, the quality of surgical margins obtained in our study did not depend on the most important risk factors for the lack of radical tumor resection in patients undergoing BCT. As it was demonstrated in the previous studies, they include lobular carcinoma, size of primary lesions (over 2 cm), presence of the HER2 receptor, patient’s age, and BMI^5,6,19,20^. Therefore, it can be assumed that the most important factor influencing the number of reoperations was the use of the intraoperative analysis of cancer mass resection margins. However, as it was demonstrated, the percentage of reoperations was similar in both studied groups.

As the research of Rubio *et al*.^12^ and Edwards *et al*.^21^ demonstrated, factors influencing the percentage of reoperations after BCT also include the weight and the volume of the resected surgical specimen. However, resection of too much tissue may be significantly decisive for the achieved aesthetic treatment outcome, at the same time, requiring the use of appropriate surgical techniques^22^. Yet, as it has already been mentioned, these parameters were not determined in the studied group of patients.

All patients post non-radical tumor resection were qualified for reoperation on a basis of the same criteria. Nonetheless, in Group II, patients were statistically more frequently treated by mastectomy (9.7% vs 5.9% – p = 0.0202). The final choice of therapeutic approach (requadrantectomy or mastectomy) was left to the patient’s individual decision. First of all, the patients were informed about a possibility of the BCT reoperation, and about a risk of late complications post mastectomy^23–25^.

The use of the intraoperative FS pathologic analysis of surgical margins at our center resulted in significantly longer duration of surgical intervention (p < 0.0001). It also required the assistance of a pathologist experienced and skilled in such analyses. Bolger *et al*. claim that the intraoperative analysis of a tissue sample does not contribute to excessive prolongation of BCT^26^. However, according to other authors and to our own previous findings, the use of the intraoperative analysis does significantly increase duration of the surgical procedure^8–11^.

Although the intraoperative pathologic analysis of excision margins was not used in Group II, the width of tumor resection margins in these patients did not increase. Thus, the lack of simultaneous analysis of the extent of the surgery did not resulted in the use of excessive margins in the primary lumpectomy, when compared to patients from Group I.

In our clinical material, the BCT intervention was most frequently possible in patients with luminal A breast cancer (62.9% of analyzed cases), while BCT was the least frequently performed in patients diagnosed as HER2-positive (3.8% of women qualified for the study). The obtained data is complaint with results presented in other studies^27,28^.

The low sensitivity of the FS analysis demonstrated in our study differs significantly from that reported by other authors. According to results obtained by Olson *et al*.^29^ and Dener *et al*.^30^, it can reach 73.1% to 100%.

Keeping in mind limitations of the intraoperative margin analysis (particularly, the loss of tissue, which cannot be further evaluated in the final analysis), it is necessary to determine the validity of a total abandonment of this approach to the pathologic analysis. As shown in our clinical material, the reoperation rate post BCT due to a non-radical resection slightly decreases if the intraoperative pathologic analysis is performed (p = 0.2373). On the other hand, mainly due to the limited sensitivity of the intraoperative FS method, the reoperation was necessary in 72 (14.3%) patients. Therefore, maybe a routine replacement of the FS analysis with another, less complex diagnostic method would be a reasonable approach. The intraoperative MMG assessment of a specimen following BCT may be useful for this purpose.

It appears that of various available methods^7,12^, macroscopic margin assessment (MMA) performed by a pathologist is a promising solution for this issue. During this assessment, the width of margins for the primary lesion resection must be determined macroscopically. The result below 5 mm implies a need for supplementary resection in an appropriate direction and during the same surgical intervention^26,31^. As shown by Bolger *et al*., the use of MMA in patients undergoing BCT resulted in a decrease in the reoperation rate (to 26% vs. 34% without this method)^22^.

A less popular method for improving the quality of BCT procedure is a simultaneous removal of additional cavity shave margins (CSM). It is performed in all directions (superior, inferior, lateral, medial, anterior and posterior margins), with a minimum sample width of 1 cm. It allows reducing the reoperation rate after BCT; however, there is a possibility of a negative aesthetic outcome of such treatment^26,32^.

## Conclusions

The use of the intraoperative pathologic analysis by the frozen section (FS) method in patients undergoing BCT leads to a number of clinical consequences. The most important problem associated with the use of FS is its low sensitivity. It may be a main reason for its limited popularity and a failure to bring the use of it into common practice.

The positive results of this diagnostic method include a reduction in the total number of reoperations performed due to presence of cancer tissue in surgical margins after local resection of the primary lesion (in our study, with no statistically significant differences vs the group of patients in whom the final pathologic analysis of the BCT specimen was performed). Thus, the benefits of FS include a marginal reduction in the total number of reoperations performed.

It should also be emphasised that the use of FS results in several organizational (a need for constant presence of a pathologist skilled in performing of FS) and economic (additional diagnostics-related costs) issues. It also statistically significantly increases the overall duration of the surgical procedure.

Abandonment of the intraoperative pathologic analysis of surgical margins requires application of an alternate method for their verification, according to diagnostic capacities of a given center. Limiting this examination to the gross clinical evaluation of the lumpectomy specimen performed by the operating surgeon is insufficient. This implies a necessity for selection and the consistent use of one of methods available in this area. Here, the new methods for intraoperative verification of surgical margins post BCT (e.g., ClearEdge, MarginProbe) should also be noted. However, their use is not sufficiently wide spread yet.

## Data Availability

This study is a retrospective analysis based on clinical data from the hospital cancer registry. Data from the hospital cancer registry provided anonymised patient data. Therefore, informed consent from patients were not required for this study.
